# Supplementary material for: Heparin-based hydrogel scaffolding alters the transcriptomic profile and increases the chemoresistance of MDA-MB-231 triple-negative breast cancer cells
Source: Biomater Sci. 2020 Feb 13;8(10):2786–96. doi: 10.1039/c9bm01481k (PMC7497406; doi:10.1039/c9bm01481k)
Supplement: Supplementary file 2 [file BM-008-C9BM01481K-s002.zip › Supplementary File 4/HP-B hydrogel + EGF (s)vControl/Pathways/my_analysis.Gsea.1545201170987/HALLMARK_HYPOXIA.html]

Details for gene set HALLMARK\_HYPOXIA[GSEA]

|  || Dataset | expr.class.cls#HYDEGF\_versus\_CONTROL.class.cls#HYDEGF\_versus\_CONTROL\_repos |
| Phenotype | class.cls#HYDEGF\_versus\_CONTROL\_repos |
| Upregulated in class | CONTROL |
| GeneSet | HALLMARK\_HYPOXIA |
| Enrichment Score (ES) | -0.47891262 |
| Normalized Enrichment Score (NES) | -2.2573037 |
| Nominal p-value | 0.0 |
| FDR q-value | 0.0 |
| FWER p-Value | 0.0 |
Table: GSEA Results Summary

  

Fig 1: Enrichment plot: HALLMARK\_HYPOXIA      
 Profile of the Running ES Score & Positions of GeneSet Members on the Rank Ordered List

  

| PROBE | DESCRIPTION (from dataset) | GENE SYMBOL | GENE\_TITLE | RANK IN GENE LIST | RANK METRIC SCORE | RUNNING ES | CORE ENRICHMENT || 1 | ATF3 | na |  |  | 473 | 2.154 | -0.0128 | No |
| 2 | PHKG1 | na |  |  | 725 | 1.894 | -0.0154 | No |
| 3 | PYGM | na |  |  | 842 | 1.788 | -0.0115 | No |
| 4 | PRKCA | na |  |  | 865 | 1.773 | -0.0028 | No |
| 5 | DTNA | na |  |  | 946 | 1.718 | 0.0026 | No |
| 6 | SCARB1 | na |  |  | 994 | 1.690 | 0.0096 | No |
| 7 | PPP1R15A | na |  |  | 1051 | 1.653 | 0.0159 | No |
| 8 | EXT1 | na |  |  | 1070 | 1.642 | 0.0241 | No |
| 9 | ADORA2B | na |  |  | 1353 | 1.493 | 0.0176 | No |
| 10 | EGFR | na |  |  | 1395 | 1.470 | 0.0237 | No |
| 11 | ENO3 | na |  |  | 1899 | 1.295 | 0.0045 | No |
| 12 | FOS | na |  |  | 2215 | 1.207 | -0.0053 | No |
| 13 | ILVBL | na |  |  | 2247 | 1.197 | -0.0003 | No |
| 14 | TPST2 | na |  |  | 2431 | 1.145 | -0.0035 | No |
| 15 | CHST3 | na |  |  | 2546 | 1.116 | -0.0032 | No |
| 16 | DDIT3 | na |  |  | 2748 | 1.066 | -0.0078 | No |
| 17 | NEDD4L | na |  |  | 2847 | 1.042 | -0.0072 | No |
| 18 | F3 | na |  |  | 2957 | 1.016 | -0.0072 | No |
| 19 | NDST1 | na |  |  | 2964 | 1.014 | -0.0019 | No |
| 20 | HOXB9 | na |  |  | 3002 | 1.003 | 0.0018 | No |
| 21 | STC2 | na |  |  | 3009 | 1.002 | 0.0071 | No |
| 22 | IRS2 | na |  |  | 3062 | 0.988 | 0.0099 | No |
| 23 | DUSP1 | na |  |  | 3230 | 0.954 | 0.0064 | No |
| 24 | CTGF | na |  |  | 3508 | 0.903 | -0.0031 | No |
| 25 | GPC1 | na |  |  | 3618 | 0.882 | -0.0039 | No |
| 26 | ANGPTL4 | na |  |  | 4183 | 0.784 | -0.0291 | No |
| 27 | SIAH2 | na |  |  | 4404 | 0.748 | -0.0365 | No |
| 28 | MAFF | na |  |  | 4719 | 0.703 | -0.0491 | No |
| 29 | COL5A1 | na |  |  | 4746 | 0.696 | -0.0466 | No |
| 30 | TIPARP | na |  |  | 4770 | 0.692 | -0.0439 | No |
| 31 | CHST2 | na |  |  | 5270 | 0.610 | -0.0667 | No |
| 32 | BCL2 | na |  |  | 5367 | 0.594 | -0.0684 | No |
| 33 | B3GALT6 | na |  |  | 5471 | 0.580 | -0.0706 | No |
| 34 | JUN | na |  |  | 5642 | 0.555 | -0.0764 | No |
| 35 | NR3C1 | na |  |  | 5797 | 0.533 | -0.0815 | No |
| 36 | FOXO3 | na |  |  | 6580 | 0.427 | -0.1202 | No |
| 37 | HDLBP | na |  |  | 7279 | 0.332 | -0.1551 | No |
| 38 | SLC37A4 | na |  |  | 7422 | 0.314 | -0.1608 | No |
| 39 | JMJD6 | na |  |  | 8239 | 0.216 | -0.2024 | No |
| 40 | EDN2 | na |  |  | 8553 | 0.181 | -0.2179 | No |
| 41 | PPP1R3C | na |  |  | 8877 | 0.138 | -0.2340 | No |
| 42 | PPFIA4 | na |  |  | 9532 | 0.071 | -0.2680 | No |
| 43 | TPBG | na |  |  | 9534 | 0.070 | -0.2677 | No |
| 44 | GCNT2 | na |  |  | 9548 | 0.069 | -0.2680 | No |
| 45 | GRHPR | na |  |  | 9778 | 0.039 | -0.2798 | No |
| 46 | AKAP12 | na |  |  | 10058 | 0.011 | -0.2944 | No |
| 47 | PFKL | na |  |  | 10128 | 0.001 | -0.2980 | No |
| 48 | HMOX1 | na |  |  | 10412 | -0.037 | -0.3127 | No |
| 49 | PFKP | na |  |  | 10539 | -0.041 | -0.3190 | No |
| 50 | CITED2 | na |  |  | 10743 | -0.071 | -0.3293 | No |
| 51 | PGM2 | na |  |  | 10868 | -0.089 | -0.3353 | No |
| 52 | SLC6A6 | na |  |  | 10932 | -0.099 | -0.3381 | No |
| 53 | KDM3A | na |  |  | 11064 | -0.116 | -0.3443 | No |
| 54 | PIM1 | na |  |  | 11181 | -0.119 | -0.3497 | No |
| 55 | CAV1 | na |  |  | 11391 | -0.145 | -0.3599 | No |
| 56 | FBP1 | na |  |  | 11864 | -0.213 | -0.3835 | No |
| 57 | SDC3 | na |  |  | 11878 | -0.214 | -0.3830 | No |
| 58 | FOSL2 | na |  |  | 12155 | -0.238 | -0.3962 | No |
| 59 | HK1 | na |  |  | 12315 | -0.263 | -0.4031 | No |
| 60 | TGFB3 | na |  |  | 12352 | -0.267 | -0.4035 | No |
| 61 | INHA | na |  |  | 12498 | -0.286 | -0.4095 | No |
| 62 | PLAC8 | na |  |  | 13041 | -0.347 | -0.4360 | No |
| 63 | SLC2A1 | na |  |  | 13117 | -0.356 | -0.4380 | No |
| 64 | HS3ST1 | na |  |  | 13132 | -0.358 | -0.4367 | No |
| 65 | ZNF292 | na |  |  | 13183 | -0.366 | -0.4373 | No |
| 66 | NDST2 | na |  |  | 13199 | -0.371 | -0.4360 | No |
| 67 | CSRP2 | na |  |  | 13424 | -0.407 | -0.4455 | No |
| 68 | GPI | na |  |  | 13512 | -0.415 | -0.4477 | No |
| 69 | MAP3K1 | na |  |  | 13524 | -0.417 | -0.4460 | No |
| 70 | CDKN1C | na |  |  | 13565 | -0.423 | -0.4457 | No |
| 71 | KLF6 | na |  |  | 13608 | -0.426 | -0.4456 | No |
| 72 | CP | na |  |  | 13757 | -0.449 | -0.4508 | No |
| 73 | GYS1 | na |  |  | 13805 | -0.459 | -0.4507 | No |
| 74 | ZFP36 | na |  |  | 13904 | -0.472 | -0.4532 | No |
| 75 | HEXA | na |  |  | 13975 | -0.484 | -0.4542 | No |
| 76 | STBD1 | na |  |  | 14033 | -0.493 | -0.4545 | No |
| 77 | PLAUR | na |  |  | 14166 | -0.503 | -0.4586 | No |
| 78 | BCAN | na |  |  | 14416 | -0.540 | -0.4686 | No |
| 79 | GAA | na |  |  | 14430 | -0.545 | -0.4663 | No |
| 80 | VHL | na |  |  | 14433 | -0.546 | -0.4633 | No |
| 81 | PDK1 | na |  |  | 14692 | -0.588 | -0.4736 | No |
| 82 | ENO2 | na |  |  | 14703 | -0.591 | -0.4708 | No |
| 83 | AMPD3 | na |  |  | 14721 | -0.592 | -0.4684 | No |
| 84 | RRAGD | na |  |  | 14803 | -0.604 | -0.4693 | No |
| 85 | CDKN1A | na |  |  | 14882 | -0.616 | -0.4700 | No |
| 86 | XPNPEP1 | na |  |  | 14895 | -0.618 | -0.4671 | No |
| 87 | GALK1 | na |  |  | 14903 | -0.619 | -0.4640 | No |
| 88 | NAGK | na |  |  | 14978 | -0.629 | -0.4644 | No |
| 89 | PFKFB3 | na |  |  | 15066 | -0.646 | -0.4654 | No |
| 90 | ETS1 | na |  |  | 15068 | -0.646 | -0.4618 | No |
| 91 | MT1E | na |  |  | 15125 | -0.658 | -0.4611 | No |
| 92 | EFNA3 | na |  |  | 15291 | -0.685 | -0.4659 | No |
| 93 | ANKZF1 | na |  |  | 15352 | -0.697 | -0.4652 | No |
| 94 | SLC25A1 | na |  |  | 15598 | -0.742 | -0.4739 | No |
| 95 | ADM | na |  |  | 15599 | -0.743 | -0.4698 | No |
| 96 | CDKN1B | na |  |  | 15677 | -0.758 | -0.4696 | No |
| 97 | PDGFB | na |  |  | 15834 | -0.789 | -0.4734 | No |
| 98 | NFIL3 | na |  |  | 15878 | -0.797 | -0.4712 | No |
| 99 | HK2 | na |  |  | 15926 | -0.806 | -0.4691 | No |
| 100 | IGFBP1 | na |  |  | 16061 | -0.836 | -0.4715 | No |
| 101 | DDIT4 | na |  |  | 16203 | -0.871 | -0.4740 | Yes |
| 102 | TPD52 | na |  |  | 16281 | -0.893 | -0.4731 | Yes |
| 103 | WSB1 | na |  |  | 16370 | -0.916 | -0.4726 | Yes |
| 104 | PGF | na |  |  | 16446 | -0.936 | -0.4713 | Yes |
| 105 | ALDOA | na |  |  | 16587 | -0.967 | -0.4733 | Yes |
| 106 | MT2A | na |  |  | 16590 | -0.967 | -0.4680 | Yes |
| 107 | TES | na |  |  | 16614 | -0.974 | -0.4637 | Yes |
| 108 | PPARGC1A | na |  |  | 16670 | -0.992 | -0.4611 | Yes |
| 109 | TGFBI | na |  |  | 16713 | -1.002 | -0.4577 | Yes |
| 110 | BHLHE40 | na |  |  | 16798 | -1.027 | -0.4564 | Yes |
| 111 | TGM2 | na |  |  | 16812 | -1.030 | -0.4513 | Yes |
| 112 | PNRC1 | na |  |  | 16832 | -1.038 | -0.4465 | Yes |
| 113 | TNFAIP3 | na |  |  | 16896 | -1.053 | -0.4439 | Yes |
| 114 | RBPJ | na |  |  | 16927 | -1.063 | -0.4396 | Yes |
| 115 | CXCR4 | na |  |  | 17068 | -1.103 | -0.4408 | Yes |
| 116 | SLC2A5 | na |  |  | 17113 | -1.116 | -0.4369 | Yes |
| 117 | SRPX | na |  |  | 17235 | -1.154 | -0.4368 | Yes |
| 118 | LOX | na |  |  | 17366 | -1.203 | -0.4369 | Yes |
| 119 | EFNA1 | na |  |  | 17471 | -1.245 | -0.4354 | Yes |
| 120 | PGM1 | na |  |  | 17488 | -1.254 | -0.4292 | Yes |
| 121 | KLF7 | na |  |  | 17603 | -1.294 | -0.4280 | Yes |
| 122 | SDC2 | na |  |  | 17642 | -1.310 | -0.4227 | Yes |
| 123 | SAP30 | na |  |  | 17712 | -1.340 | -0.4188 | Yes |
| 124 | BTG1 | na |  |  | 17748 | -1.358 | -0.4130 | Yes |
| 125 | TPI1 | na |  |  | 17749 | -1.359 | -0.4055 | Yes |
| 126 | HSPA5 | na |  |  | 17766 | -1.367 | -0.3987 | Yes |
| 127 | VLDLR | na |  |  | 17779 | -1.371 | -0.3916 | Yes |
| 128 | ISG20 | na |  |  | 17840 | -1.410 | -0.3869 | Yes |
| 129 | P4HA2 | na |  |  | 17913 | -1.447 | -0.3826 | Yes |
| 130 | IDS | na |  |  | 18220 | -1.611 | -0.3897 | Yes |
| 131 | IL6 | na |  |  | 18227 | -1.619 | -0.3810 | Yes |
| 132 | SELENBP1 | na |  |  | 18289 | -1.668 | -0.3748 | Yes |
| 133 | PAM | na |  |  | 18328 | -1.699 | -0.3673 | Yes |
| 134 | CCNG2 | na |  |  | 18336 | -1.703 | -0.3582 | Yes |
| 135 | UGP2 | na |  |  | 18385 | -1.741 | -0.3510 | Yes |
| 136 | P4HA1 | na |  |  | 18626 | -1.958 | -0.3527 | Yes |
| 137 | GLRX | na |  |  | 18639 | -1.972 | -0.3423 | Yes |
| 138 | IGFBP3 | na |  |  | 18665 | -2.008 | -0.3324 | Yes |
| 139 | TMEM45A | na |  |  | 18692 | -2.035 | -0.3224 | Yes |
| 140 | FAM162A | na |  |  | 18708 | -2.062 | -0.3116 | Yes |
| 141 | SDC4 | na |  |  | 18719 | -2.074 | -0.3006 | Yes |
| 142 | S100A4 | na |  |  | 18812 | -2.210 | -0.2931 | Yes |
| 143 | AK4 | na |  |  | 18825 | -2.239 | -0.2812 | Yes |
| 144 | LXN | na |  |  | 18837 | -2.262 | -0.2691 | Yes |
| 145 | NDRG1 | na |  |  | 18853 | -2.277 | -0.2572 | Yes |
| 146 | PDK3 | na |  |  | 18854 | -2.278 | -0.2445 | Yes |
| 147 | GBE1 | na |  |  | 18855 | -2.282 | -0.2317 | Yes |
| 148 | KDELR3 | na |  |  | 18881 | -2.328 | -0.2201 | Yes |
| 149 | PLIN2 | na |  |  | 18894 | -2.342 | -0.2076 | Yes |
| 150 | STC1 | na |  |  | 18901 | -2.351 | -0.1948 | Yes |
| 151 | ERRFI1 | na |  |  | 18921 | -2.402 | -0.1824 | Yes |
| 152 | ATP7A | na |  |  | 18940 | -2.432 | -0.1697 | Yes |
| 153 | CASP6 | na |  |  | 18952 | -2.470 | -0.1565 | Yes |
| 154 | BNIP3L | na |  |  | 18957 | -2.475 | -0.1429 | Yes |
| 155 | MXI1 | na |  |  | 18988 | -2.556 | -0.1302 | Yes |
| 156 | SLC2A3 | na |  |  | 19022 | -2.648 | -0.1171 | Yes |
| 157 | ALDOC | na |  |  | 19062 | -2.788 | -0.1036 | Yes |
| 158 | WISP2 | na |  |  | 19077 | -2.817 | -0.0886 | Yes |
| 159 | RORA | na |  |  | 19128 | -3.064 | -0.0741 | Yes |
| 160 | CA12 | na |  |  | 19153 | -3.311 | -0.0569 | Yes |
| 161 | PGK1 | na |  |  | 19154 | -3.315 | -0.0384 | Yes |
| 162 | PRDX5 | na |  |  | 19161 | -3.438 | -0.0195 | Yes |
| 163 | KLHL24 | na |  |  | 19182 | -3.826 | 0.0008 | Yes |
Table: GSEA details [plain text format]

  

Fig 2: HALLMARK\_HYPOXIA      
 Blue-Pink O' Gram in the Space of the Analyzed GeneSet

  

Fig 3: HALLMARK\_HYPOXIA: Random ES distribution      
 Gene set null distribution of ES for **HALLMARK\_HYPOXIA**
